# Enhanced Ablative Performance of Additively Manufactured Thermoplastic Composites for Lightweight Thermal Protection Systems (TPS)

**DOI:** 10.3390/polym17182462

**Published:** 2025-09-11

**Authors:** Teodor Adrian Badea, Lucia Raluca Maier, Alexa-Andreea Crisan

**Affiliations:** Composite Materials Laboratory for Aeronautical Field, Romanian Research & Development Institute for Gas Turbines—COMOTI, 220D Iuliu Maniu Av., 061126 Bucharest, Romania; teodor.badea@comoti.ro (T.A.B.); alexa.crisan@comoti.ro (A.-A.C.)

**Keywords:** ablative TPS, additive manufacturing, FRTPC, surface coating, oxyacetylene torch

## Abstract

The research investigated the potential of five novel additively manufactured (AM) fiber-reinforced thermoplastic composite (FRTPC) configurations as alternatives for ablative thermal protection system (TPS) applications. The thermal stability and ablative behavior of ten samples developed via fused deposition modeling (FDM) three-dimensional (3D) printing out of fire-retardant thermoplastics were investigated using an in-house oxyacetylene torch bench. All samples featured an innovative internal thermal management architecture with three air chambers. Furthermore, the enhancement of thermal benefits was achieved through several approaches: ceramic coating, mechanical hybridization, or continuous fiber reinforcement. For each configuration, two samples were exposed to flame at 1450 ± 50 °C for 30 s and 60 s, respectively, with the front surface subjected to direct exposure at a distance of 100 mm during the ablation tests. Internal temperatures recorded at two back-side contact points remained below 50 °C, well under the 180 °C maximum allowable back-face temperature for TPS during testing. Continuous reinforced configurations 4 and 5 displayed higher thermal stability the lowest values in terms of thickness, mass loss, and recession rates. Both configurations showed half of the weight losses measured for the other tested configurations, ranging from approximately 5% (30 s) to 10–12% (60 s), confirming the trend observed in the thickness loss measurements. However, continuous glass-reinforced configuration 5 exhibited the lowest weight loss values for both exposure durations, benefiting from its non-combustible nature, low thermal conductivity, and high abrasion resistance intrinsic characteristics. In particular, the Al_2_O_3_ surface coated configuration 1 showed a mass loss comparable to reinforced configurations, indicating that an enhanced surface coat adhesion could provide a potential benefit. A key outcome of the study was the synergistic effect of the novel air chamber architecture, which reduces thermal conductivity by forming small internal air pockets, combined with the continuous front-wall fiber reinforcement functioning as a thermal and abrasion barrier. This remains a central focus for future research and optimization.

## 1. Introduction

Thermal Protection Systems (TPS) protect vehicles from extreme heat loads during hypersonic or atmospheric re-entry flights. Traditional ablative TPS materials, such as carbon–carbon (C/C) and carbon–phenolic composites (CPCs), are often enhanced with ultra-high temperature ceramics (UHTC) coatings to improve high-temperature performance [1]. In parallel, plasma-assisted materials (PAMs) provide lightweight thermal protection for extreme environments, supporting advanced propulsion systems across various rocket types [2,3].

Comparative studies have identified super lightweight ablator (SLA)-561V, phenolic impregnated carbon ablator (PICA), AVCOAT, silicone impregnated reusable ceramic ablator (SIRCA), and PICA-X as the most efficient ablative materials [4]. Recent developments demonstrate that hybrid TPSs, combining flexible insulation with phase change materials (PCM), can enhance thermal insulation and reduce system thickness, achieving up to 2.73× higher structural efficiency than traditional designs, although ablation resistance may decrease under prolonged re-entry trajectories [5].

Material innovations continue to advance thermal, mechanical, and fire performance. For instance, poly-DDPM-coated rigid polyurethane foam (RPUF) with expandable graphite (EG), applied via brush painting, improved limiting oxygen index (LOI) from 18 to 32.3, upgraded UL-94 flame rating to V-0, reduced peak heat release rate (PHRR) and total smoke production (TSP) by 55% and 59%, and increased compressive strength by 10%, with minimal weight gain (8.5 mg/cm^2^) [6]. Similarly, a ~10 μm thermoset polymer coating with ceramic particles, applied via liquid spray on material extrusion additive manufacturing (MEAM)-fabricated polyphenylene sulfide (PPS)/50 wt% carbon fiber composites, enhanced abrasion resistance (89%), reduced vacuum loss (95%), and improved demolding force (53.2%) and static (40%) and kinetic (46.4%) friction [7].

Advanced hybrid coatings comprising graphene material (GM), nano black phosphorus–SiO_2_ aerogel, and phosphorus–acrylic polymer achieved UL-94 V-0, LOI 45%, self-extinguishing > 1100 °C, and reduced PHRR and TSP by ~50%, with strong substrate adhesion [8]. High-temperature composite 3D printing (HC3DP), a novel material extrusion method, demonstrated superior thermal resistance, lower U-values, reduced material consumption, faster fabrication, and additional benefits such as lightweight, translucent facades and aerogel integration [9].

Polyamide (PA6) composites with halogen-free flame retardants and hybrid metal synergists met EN45545 R22 HL3 standards (LOI ≥ 32%, smoke density ≤ 300, critical insulation temperature (CIT) ≤ 1.5, UL-94 V-0, glass wool fire index (GWFI) 960 °C), demonstrating improved fire and thermal performance [10]. Cerakwool, coated with toughened uni-directional fabric interface (TUFI), showed no ablation after high-velocity oxygen fuel (HVOF) testing at 0.65–1 MW/m^2^, with a 200 °C stagnation point rise and internal temperatures below 50 °C at 40 mm depth, staying within the 180 °C back-face limit [11].

Experimental validations such as Kentucky Re-Entry Probe Experiment 1 (KREPE1, 2021) and KREPE2 (2023) confirmed the suitability and stability of 3D-printed cyanate ester and phenolic dual-layer ablative shields via arcjet tests [12]. UHTCs, including ZrB_2_, HfB_2_, ZrC, HfC, TaC, and high-entropy variants, often enhanced with SiC or MoSi_2_ coatings, provide exceptional thermal and mechanical properties, with tailored microporosity improving thermal shock resistance and fracture strength [13,14]. For example, a 6 mol% Sm_2_O_3_-stabilized t′-ZrO_2_ coating, applied via atmospheric plasma spray (APS), improved C/C ablation resistance under ~390 W/cm^2^ heat flux, reducing mass and linear ablation rates by ~71% and ~94%, due to high thermal shock resistance and mechanical adhesion [15].

NASA Ames employs arc jet testing, computational fluid dynamics (CFD), and laser diagnostics to improve TPS test-flight correlation and reduce boundary uncertainties [16]. An axisymmetric CFD study using Ansys Fluent with a non-premixed combustion model found the k-ε RNG turbulence model best matched oxyacetylene torch ablation test data, with heat flux deviations under 10% [17]. Plasma wind tunnel tests of Zuram, Asterm, and Calcarb ablators under identical PWK1 conditions revealed Asterm achieved the highest heat flux mitigation and lowest recession rates due to effective pyrolysis and boundary layer interactions [18]. Spectral emissivity, reflectivity, and transmissivity measurements of carbon fiber reinforced polymer (CFRP) and glass fiber reinforced polymer (GFRP) composites validated thermography-based defect quantification [19], while oxyacetylene torch (OAT) testing confirmed thermal diffusivity of CPCs [3].

The newly developed ablative Thermal Protection Systems (TPSs) are single-use, sacrificial materials that degrade during operation, forming a protective char layer and absorbing heat. These systems are essential for safeguarding vehicles during hypersonic flight and atmospheric re-entry. This study demonstrates enhanced lightweight thermal performance through a combination of ceramic-coated ablators, hybrid or continuous fiber-reinforced structures, and a novel 3D-printed air chamber architecture designed to minimize thermal conductivity and convective heat transfer. By integrating advanced coatings, fiber reinforcement, and additive manufacturing, the proposed TPS configurations achieve higher structural efficiency, reduced weight, and superior heat resistance, representing a significant advancement beyond the state-of-the-art in ablative thermal management. These innovations provide a promising pathway for next-generation aerospace applications.

## 2. Materials and Methods

### 2.1. Materials

**Figure 1:**
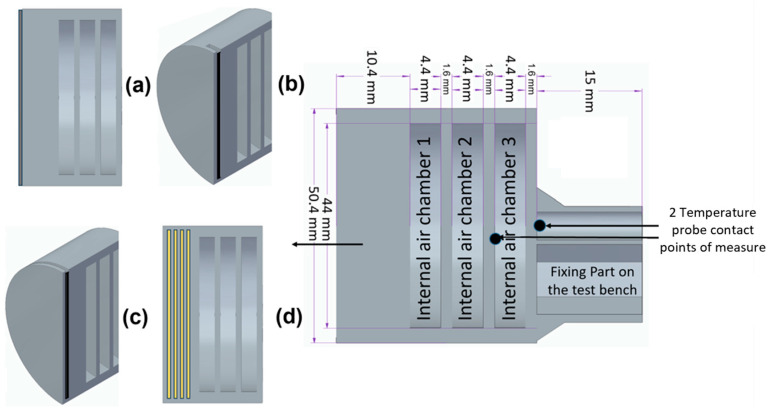
Illustration of the newly developed ablative TPS configurations incorporating the innovative internal air chamber architecture: (**a**) configuration 1 Al_2_O_3_ powder surface coating; (**b**) configuration 2 slot filled subsequently with 5 g of Al_2_O_3_ powder; (**c**) configuration 3 slot filled with 2 mm thickness CFRP laminate disk; (**d**) configurations 4 and 5 continuously reinforced with carbon and, respectively, glass fibers.

The newly developed 3D-printed ablative TPS configurations were developed via FDM 3D printing technology out of fire-retardant Onyx FR-V0 material, and all integrating an internal air chamber concept architecture for thermal management. The proposed novel tri-chamber internal architecture (Figure 1) functions on the fundamental insulation principle of subdividing air volumes, thereby diminishing convective and radiative heat transfer, reducing thermal conductivity, and concurrently offering mass efficiency advantages. The selected bulk structural material, Onyx FR-V0, is the flame-retardant variant of Onyx. Onyx is a nylon (polyamide 6) composite reinforced with 10% to 20% short carbon fibers by volume, as reported in [20], while image analysis conducted by Sauer [21] revealed a chopped fiber content of approximately 9% within the matrix phase. Onyx FR achieved a V-0 rating on the UL94 flammability test while possessing similar mechanical properties to Onyx, having a 1.2 g/cm^3^ density a heat deflection temperature of 145 °C. Onyx has excellent properties, including lightweight mechanical properties, strong thermal stability, and resistance to ultraviolet (UV) radiation and chemicals. When reinforced with continuous carbon fibers, Onyx can reach strengths comparable to 6061-T6 aluminum, as fiber lengths, mainly aligned with the deposition direction, range from 7.035 to 44.58 μm [22].

**Figure 2:**
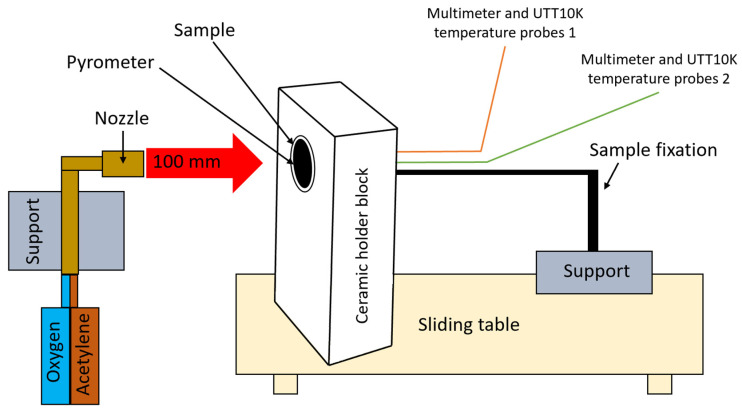
Oxyacetylene torch test bench setup (OTB ablation testing).

Furthermore, enhanced fire resistance was enabled through three different additional approaches: Al_2_O_3_ powder surface coating (Figure 1a), mechanical hybridization by 5 g Al_2_O_3_ powder-filled slot (Figure 1b) or 2 mm thickness CFRP laminate disk slot integration (Figure 1c), and, respectively, frontal wall continuous carbon and, respectively, glass fiber reinforcement (Figure 1d). Under identical testing conditions, two specimens for each configuration, exposed to 30 s and 60 s of flame, respectively, were evaluated on a custom-designed oxyacetylene torch test rig. Depending on ceramic coating, added cured carbon disk at flame interface, and reinforcement type and morphology (short discontinuous or long continuous; carbon-carbon or carbon-glass), the initial mass of each tested composite specimen varied between approximately 40 g and 50 g.

Table 1 delineates all ten tested samples of the developed composite ablative TPS fabricated full-infill via FDM additive manufacturing using Onyx FR V0. As previously mentioned, the structural architectural design of all tested samples incorporates air chambers with apertures facilitating external medium exchange, under which vacuum conditions eliminate conductive and convective heat transfer, leaving radiation as the sole mechanism. The thermal conductivity of air exhibits a slight increase with rising temperature but diminishes under reduced pressure.

Configuration 1 involved post-processing via ceramic surface modification, applying thermally integrated Al_2_O_3_ particulates mechanically affixed to the flame-impinged surface. Configuration 2 incorporated a 47.2 mm-diameter, 1.5 mm-thick slot, which was subsequently filled with 5 g of Al_2_O_3_ powder. For Configurations 1 and 2, a hard brown alumina powder (>95.5% Al_2_O_3_; Mohs hardness 9.0; density 3.9 g/cm^3^; melting point 2200 °C; 80-mesh, ~110 μm) was used. Configuration 3 included a slot into which a 2 mm-thick, thermally cured CFRP laminate disk (Hexply M49/42%/200T2×2/CHS-3K) was integrated at the flame-exposed surface to enhance thermal protection. Configurations 4 and 5 were likewise produced via FDM with full-infill structure using Onyx FR V0 and additionally continuously reinforced with carbon and glass fibers, respectively. Since the carbonaceous layer generated from the locally reinforced matrix can be easily eroded, the continuous reinforcement acts as an additional heat sink, undergoing endothermic melting and evaporation, while also limiting mechanical erosion of the surface.

### 2.2. Three-Dimensional Printing Technology

Ablative TPS samples were fabricated via FDM additive manufacturing. Onyx FR-V0 specimens were printed on a Prusa XL (Prusa Research a.s., Prague, Czech Republic) using 1.75 mm filament, while configurations 4 and 5, also using Onyx FR-V0 and reinforced with continuous glass fiber and carbon fiber, were produced on a Markforged X7, Waltham, MA, USA, employing 310 µm fiber filament. Markforged X7 printing parameters included a 275 °C nozzle temperature, unheated bed, 100 µm layer height, one shell, 0.4 mm nozzle diameter, and 100% infill. On the Prusa XL, Onyx FR-V0 samples were printed with a 290 °C nozzle and 110 °C bed. All samples shared a 0.2 mm layer height, rectilinear infill pattern, and two-shell wall structure.

### 2.3. Oxyacetylene Flame Test Rig

A purpose-built oxyacetylene torch test rig was used to perform the high-temperature oxidation testing. A schematic of the torch test facility is shown in Figure 2. Oxygen and acetylene were fed through a welding nozzle (LS14 model inner No. 4 nozzle, RHONA GCE group, Steinhausen, Switzerland) to produce a high-temperature flame (1450 ± 50 °C) oxidizing flame. For testing purposes, the samples (50 mm diameter sample, geometry features given in Figure 1) were placed in a ceramic holder featuring a 51 mm diameter circular opening for proper positioning. They were mechanically secured within a rigid support structure mounted on a mobile roller plate equipped with a locking mechanism. This setup ensured consistent positioning, allowing each sample to be placed identically within the holder. The mobile roller plate was manually advanced to the target point, aligning the flame precisely at the center of the sample’s front surface.

The distance between the nozzle and the sample was 100 mm to achieve the desired peak temperature and heating profile. Each developed configuration was tested using two samples: one exposed to flame for 30 s and the other for 60 s. Then the sample was retrieved by pulling the mobile roller plate out of the flame field, to extract the tested sample, allowing it to cool down naturally. To ensure quantitative and repeatable testing, the setup maintained a continuously lit flame throughout the entire test campaign.

During testing, the peak temperature at the center of the sample’s front surface was measured using an infrared pyrometer (Sonel DIT-500 from SONEL S.A., Swidnica, Poland). The back-face internal temperatures were recorded at two contact points located on the rear walls of the second and third air chambers, as viewed from the front of the sample (as depicted in Figure 2), using UTT10K UNI-T temperature measure probes with a range of up to 260 °C and a high accuracy of ±0.75%, both connected to two digital multimeters (UT131C from UNI-T (UNI-Trend Technology Co., Ltd., Shenzhen, China) and SMA 19 from Somogyi Elektronic Kft., Gyor, Hungary.

## 3. Results

### 3.1. Morphological Analyses

**Figure 3:**
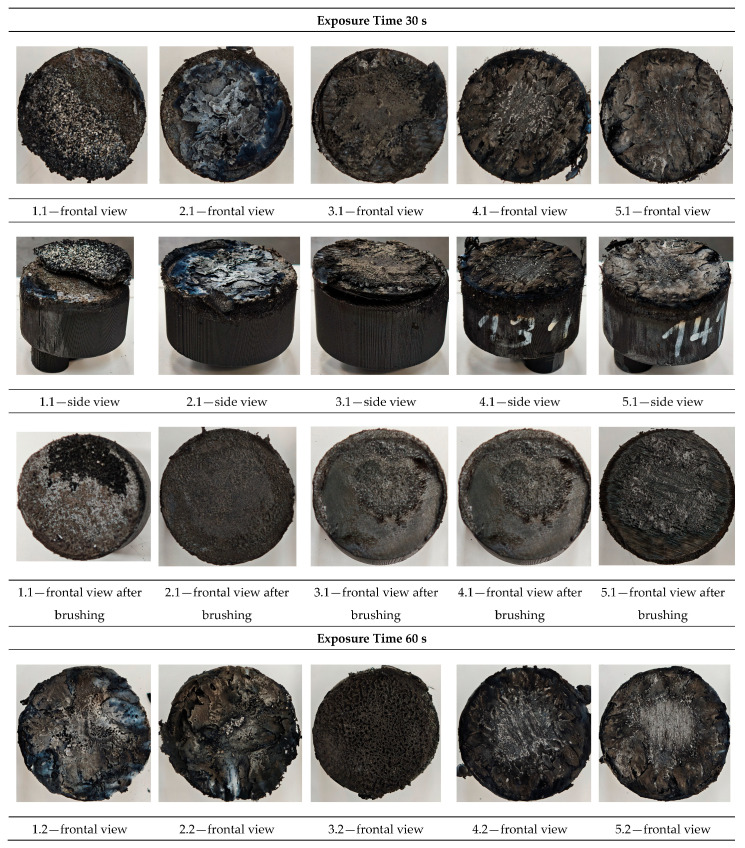

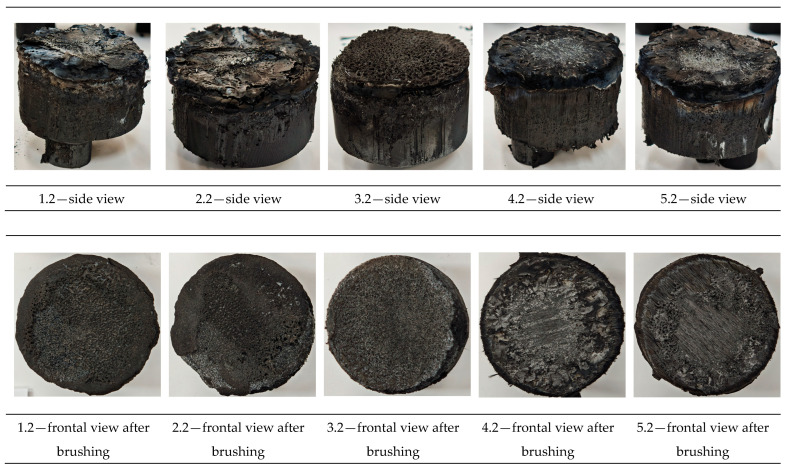
Images of the samples after OTB ablation testing: configuration/exposure time, frontal view, side view and frontal view after brushing following to char removal.

Visual inspection and morphology analysis, along with thickness measurements, were performed following the Oxyacetylene Test Bed (OTB) ablation tests. The loss of weight due to the ablation process was also evaluated. Figure 3 depicts images of the samples following OTB ablation testing, prior to and after char removal. The sample front faces can be seen to be covered with a carbon-rich char layer, with dark brown regions indicating that the expected oxidation had occurred. The visual observations indicated a mixed mechanism during ablation of dripping accompanied by charring. The brown color of the burnt surface of samples is potentially related to the presence of the metal hydroxides FR additives from the bulk thermoplastic, enhancing fire safety by reducing flammability and promoting self-extinguishment. The char layer acts as a barrier to heat transfer; its porous structure provides insulation and potentially can also re-radiate some of the absorbed heat. Then as the surface of the char layer erodes away through ablation, it carries away heat energy, further protecting the underlying material. Dissimilar to traditional ablative materials that develop a thick char layer, thermoplastic ablatives typically exhibit higher gas formation rates at lower surface temperatures and produce a comparatively thinner char layer.

The post-testing appearance of exposed surfaces of the samples was characterized by a wide and relatively broad eroded surface, while both 4 and 5 continuously reinforced configurations showed a confined central region, strongly confined under the plume of the impinging torch, revealing the fibers.

In the case of both samples of configuration 2, the 5 g of Al_2_O_3_ from the inner slot near the surface did not thermo-mechanically adhere to the beneath structural material, since the surface temperature achieved was well below its 2200 °C melting point; thus, it completely detached from the base material. A comparable behavior was observed on the configuration 1 surface-coated samples. The sample frontal view exhibits more pronounced detachment of the surface coating due to high thermal flux exposure for a longer time (60 s) compared to 30 s exposure time, indicating poor adhesion and embedded into the Onyx FR V0 melted layer adjacent to the bulk material of the Al_2_O_3_ powder layer. Likewise, cracking or debonding from the substrate was also attributed to the difference in coefficients of thermal expansion between the substrate and the coating. Once the coating was ablatively removed, it vanished to exert the protective function.

Referring to configuration 3, significant thermal erosion protection was observed on the 3.1 sample exposed for 30 s, given by the near-surface slot integrated CFRP laminate disk and especially by the 2D carbon weave. Nevertheless, the disk was completely removed on the 3.2 sample exposed for 60 s, since the CFRP disk was only integrated in the slot and no mechanical bonding was assured prior to OTB ablation testing. Configurations 4 and 5, carbon and glass, respectively, that were continuously reinforced, exhibit improved thermal behavior among all developed configurations. Both configurations were developed in order to assess the contribution of the additional continuous reinforcement phase to the thermal resistance and ablation performance of the newly developed 3D-printed TPS configurations, beyond the influence of the novel internal air chamber architectural concept. Carbon fibers have excellent thermal stability and can withstand very high temperatures (often above 3000 °C in inert atmospheres); nevertheless, when exposed to high heat, in the presence of oxygen carbon fibers can oxidize (burn), typically starting around 600 °C, oxidation leading to degradation, structure breakdown, and loss of mechanical strength. On the other hand, although glass fibers start to soften at high temperatures, typically around 800 °C, they do not readily ignite or burn and are considered non-combustible. The main component of glass, silica, is already in its highest oxidation state and, thus, does not react with oxygen, meaning it will not burn in a fire. Furthermore, although of thermoplastic nature, the matrix Onyx FR V0 embedding the fibers in different configurations (short carbon/short carbon with additional long continuous fiber reinforcement) is an FR blend. Thus, when exposed to very high temperatures, they are the first to undergo thermal degradation, melting, vaporization, or sublimation, followed by the fibers degradation. The reinforcement phase effect and its role in the thermoplastic composite burning process are, however, very complex, depending on the fiber type, its quantity, orientation, length, thermal conductivity, and size agent.

Nevertheless, it is a fact that both continuously reinforced configurations showed higher thermal stability, improved ablative behavior, lower mass loss, and lower recession rate. The 10% by volume of short carbon fiber content and additional continuous fiber frontal reinforcement (configurations 4 and 5) not only provide high strength and stiffness, but also their morphology and distribution create a thermal conductivity anisotropy that plays an important role in quickly dissipating heat away from the ablation surface in one direction (e.g., along fibers) while restricting heat flow in other directions (e.g., into the material). This helped to prevent excessive heat buildup at the surface and reduced damage. The burning mechanisms differed between bulk thermoplastic and continuously reinforced composite configurations due to fiber–matrix interactions and heat transfer dynamics. In composite materials, the interface between the matrix and the reinforcing fibers played a critical role in their combustion behavior. The reinforcing fibers influenced heat conduction anisotropically, depending on their orientation, thereby affecting the internal temperature distribution. In the initial stage, melting and dripping of the thermoplastic matrix occurred. The carbon-rich char layer formed on the sample surfaces acted as a thermal barrier but was locally eroded by flame abrasion at the center of the front surface, particularly in configurations 4 and 5, exposing the matrix–reinforcement interface. A comparison between the two reinforced configurations indicates that the type of reinforcement plays a key role in the thermal protection and ablation processes. The continuous carbon fiber reinforcement phase exhibits a higher thermal conductivity when compared to glass fibers, assuring an efficient directional heat dissipation (e.g., along the fiber alignment), simultaneously restricting heat flow in other directions (through bulk material).

Moreover, following visual analysis, the recession rate and thickness mass loss measurements were performed succeeding char layer removal by brushing the surface of tested samples.

### 3.2. Cross-Sectional Examination and Recession Rate

Surface images, cross-sectional views, and post-burning thickness measurements (of the front wall before the first air chamber, as shown in Table 2) of all tested samples were analyzed after the char layer was removed by brushing. It is important to stress that the inner walls separating the three air chambers were not affected, keeping their dimensions and structural integrity for all samples exposed to 30 and 60 s. The ablation removes material through vaporization or erosion. Cross-sectional examinations indicate a thickness loss for all tested configurations when increasing the flame time exposure from 30 s to 60 s, except for continuously carbon-reinforced configuration 4. This specific configuration revealed the formation of delamination cracks and pores at the interface between the fiber layers and the bulk Onyx FR V0 material, visible but in a lower amount also in configuration 5 (continuously glass-fiber-reinforced). The solidification cracks and trapped porosity observed at the interface result from a complex interplay of mechanisms involving solid, liquid, and vapor phases, including melting, oxidation, volatilization, and liquid flow. Specifically, the formation of these features is attributed to reactive volatile compounds produced in the decomposition zone and to water vapor generated near the rear face of the composite, both of which tend to migrate through the char layer toward the heated surface. In the case of both continuously reinforced configurations, during ablation, as the matrix degrades and then erodes, the continuous reinforcements are directly exposed to the hot stream and, thus, their temperature rises. As a result, the fibers endothermically react with the hot gas stream acting as a heat sink. During the process, part of the gases was trapped due to the composite’s low gas permeability, resulting in increased internal pressure and expansion of the material; thus, the thickness increases with time exposure for configuration 4. Such a behavior is very common in many charring ablators. By contrast, in all the other configurations, this frontal reinforcement fiber barrier is not present, the porous nature of the char layer allowing the gases to easily flow through it.

Furthermore, reinforced configurations 4 and 5 exhibit pronounced anisotropy and heterogeneity, particularly with respect to their thermal conductivity properties. The carbon fiber thermal conductivity in the direction of the parallel fiber axis is about 250 times that of the matrix (bulk material Onyx FRV0), leading to a higher heat transfer into the fiber layers and bulk material interfaces and increased volumetric ablation of the composite and internal gas generation. The carbon fibers high thermal conductivity tended to increase the in-depth penetration of the charring phenomena, and consequently, the pyrolysis gases could not escape due to the low permeability barrier impediment; thus, pores developed into cracks, and further on, the fibers suffered severe, in-depth debonding or pull-out from the matrix. The decreased pore amount observed in configuration 5 was attributed to the lower thermal conductivity of glass fibers when compared to carbon fibers.

The lack of pore formation within 1, 2, and 3 configurations, where the additional surface coating or slot-filled materials were eliminated in the first stages during the ablation tests, is due to the front thermal barrier elimination and freedom for volatile compounds pyrolysis gas to flow through the pores of the char to the ablation surface and contribute to the surface energy balance, potentially reducing the effective convective heat flow acting on the TPS.

The rate of material recession caused by ablation was calculated by measuring the loss in specimen thickness over the duration of thermal exposure. Thickness measurements were made using a Mitutoyo CD-P15P (Mitutoyo Corporation, Kawasaki, Japan) model digital vernier caliper with a measuring range of 0–150 mm and a measuring accuracy of 0.01 mm. Following testing, only the thickness of the unreacted material was taken into account. As a result, the recession measurement captures the extent of the reaction layer’s progression into the virgin material and does not include the surface char thickness. Recession is an indicator of the time duration of thermal protection, so low values are desirable. Table 2 summarizes the results of recession rates and cross-section views of all post-tested samples.

**Figure 4:**
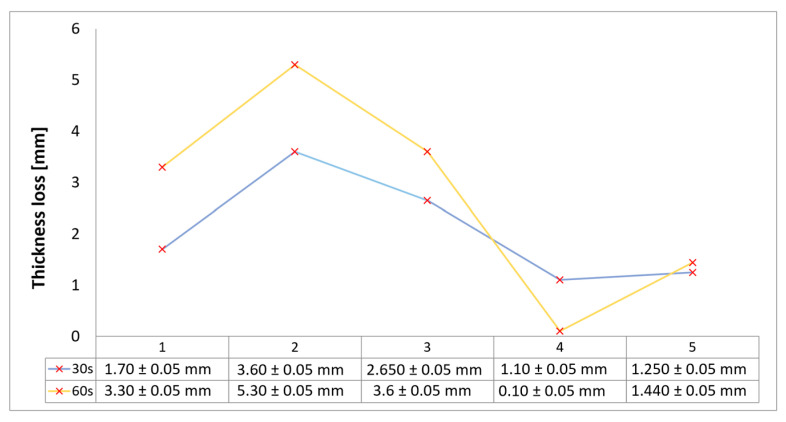
Thickness losses chart of tested configurations following char layer elimination.

With respect to the recession rate, it can be observed that both reinforced configurations 4 and 5 displayed the lowest values, with a slightly lower value for the carbon-reinforced configuration 4, indicating an increase in thermal resistance when compared to the other tested configurations. The lower recession rates are potentially attributed to both continuous carbon glass reinforcements and short/chopped carbon fibers from the Onyx FR V0 matrix that ensure an anisotropic thermal conductivity quickly dissipates heat away from the ablation surface in the fiber direction while restricting heat flow into the bulk material. In the case of carbon continuously reinforced configuration 4, the higher thermal conductivity of carbon fibers leads to an increased internal pressure and expansion of the material due to pore front formation at the interface between the fiber layers and the bulk Onyx FR V0 material. On the other hand, continuous glass fiber reinforcing the front wall of configuration 5 not only contributes to a higher structural stiffness but also through its very low thermal conductivity, acts like a frontal barrier for thermal protection due to its lower thermal conductivity. Consequently, although configuration 5 graphically indicates a higher thickness reduction when compared to carbon-reinforced configuration 4 for 60 s exposure time, glass fiber reinforced configuration 4 exhibits a higher thermal and abrasion resistance. The glass frontal layer appears denser and less impacted by volumetric expansion caused by pores, likely due to its lower thermal conductivity and greater abrasion resistance. Likewise, glass fiber thermal softening followed by degradation typically starts around 800 °C, while for carbon fibers the oxidation process begins earlier at around 600 °C. Nevertheless, overall, the above observations are consistent with the thickness losses reported in Figure 4.

Both continuous fiber reinforcements ensure a front thermal barrier during ablation tests.

Configuration 1 exhibits a slightly higher thickness loss when compared to the continuous fiber reinforced configuration and a significantly lower thickness loss when compared to configurations 2 and 3, potentially attributed to only local removal of the Al_2_O_3_ surface coating layer during ablation. In the case of configurations 2 and 3, the removal of the slot-filled materials (Al_2_O_3_ powder and, respectively, CFRP composite laminate disk) during the ablation tests led to a significant effect on thickness and mass loss.

### 3.3. Mass Loss Assessment

**Figure 5:**
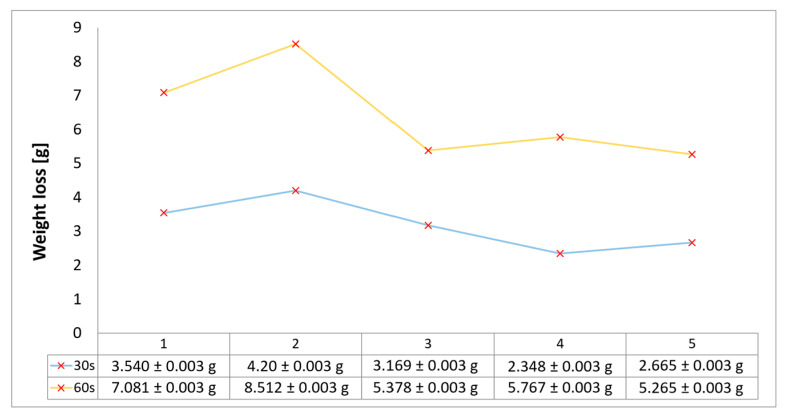
Mass loss results for OTB ablation testing of new 3D printed composite TPS configurations.

The relative ablative loss of mass (expressed as a percentage) was determined by analyzing the changes in the weight of the examined samples before and after the heat resistance tests. Mass loss was assessed after 30 s and 60 s exposure time, following char layer elimination by brushing, using a Kern PLJ 510-3M (KERN & SOHN GmbH, Balingen, Germany), model analytical balance providing a ±0.001 g precision. Mass loss values provided an indication of the time duration that ablatives can offer effective thermal protection. The results of mass loss measurements for the all tested configurations are indicated in Figure 5 and follow roughly the same trends as the results for recession rates and thickness loss. The highest ablation mass loss was recorded for configuration 2, caused by the slot-filled material Al_2_O_3_ powder removal during the ablation test, followed by configuration 1, due to the partial removal of the Al_2_O_3_ surface coating layer during ablation. Configurations 1 and 3 exhibit an both a round 8% weight loss after 30 s of exposure, whereas by increasing the time exposure to 60 s, the weight loss was higher for configuration 1 due to the Al_2_O_3_ surface coating layer (with a higher density compared to the CFRP composite laminate disk of configuration 3), both eliminated during ablation tests. The best performing weight loss values match up to those for minimal thickness loss for configurations integrating the new air chamber thermal management concept with continuous fiber reinforcement of its frontal wall. Therefore, configuration 5 presented the lower weight loss values for both exposure times, very close to results obtained for carbon continuously reinforced configuration 4, both in the range of 5% (30 s exposure) to 10–12% (60 s exposure). Overall, the weight percentage losses for all tested samples vary from 5 to 10% after 30 s of flame time exposure and increase, varying between 10 and 22%, when increasing the time exposure to 60 s.

The synergetic effect of the new air chamber concept architecture providing low thermal conductivity by creating small airspaces within the material structure, along with the continuous front wall fiber reinforcement acting like a thermal barrier, emerged as a key outcome of the study and remains a central focus for future research optimization. A clear path of optimization of configurations 4 and 5 is to increase the continuous reinforcement phase volume fraction in both frontal and radial walls, potentially decrease the thickness of the frontal wall, and additionally investigate the possibility of reducing the number of air chamber structural architecture while improving the ablative resistance. A second route for future investigation is to continue the development of hybrid configurations like 3 by combining the benefits of CFRP thermoset composites with 3D-printed TPS structures and ultimately adding a surface coating protection.

### 3.4. Temperature and Time Analysis

**Figure 6:**
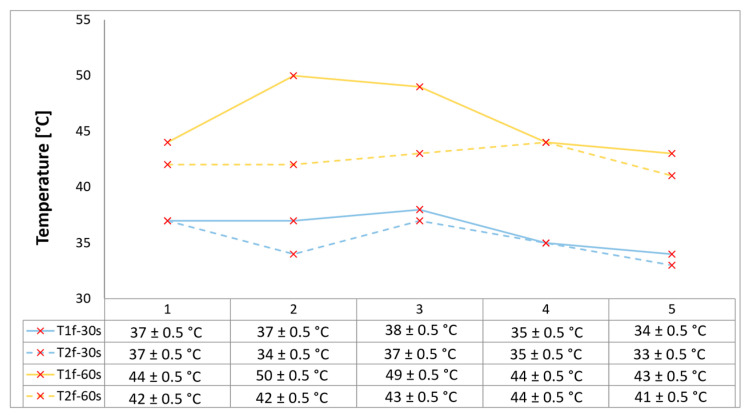
Temperature data recorded as a function of exposure time for tested OTB ablation configurations.

Since temperature is the key parameter governing ablative degradation, recording the transient internal temperature profile is essential for understanding and characterizing the thermal response of the ablator-developed materials. Two embedded temperature probes (within the ablative material at two specified distances from the surface as shown in Figure 1), each connected to a digital multimeter, continuously recorded the temperature of each sample from the start to the end of the test for both the 30 s and 60 s exposure durations. Figure 6 below provides the final recorded temperature values registered for each sample for both time exposures. In order to reduce latent distortion of the temperature measurements due to the difference in thermal diffusivity between the material and the measuring probe, the size and accuracy of ±0.75% were small, and the wires were installed perpendicular to the direction of the heat flow.

Configurations 4 and 5 exhibit a slightly lower temperature increase when compared with the other developed configurations, indicating an additional thermal protection barrier as a synergistic effect coming from the continuous fiber reinforcement (potentially heat was dissipated by re-radiation), beyond the low thermal conductivity novel internal thermal management architecture with three air chambers that has proven its benefits. Specifically, the continuously glass fiber reinforced configuration 5 exhibits the lower temperature increase due to its low thermal conductivity compared to carbon fibers.

A marginally higher temperature increase rate was recorded on 2 and 3 configurations, since both filling material slots were removed by the flame during tests, leaving the bulk material unprotected. Nevertheless, the novel three-air-chamber design provided effective thermal protection by minimizing thermal conductivity and convective heat transfer. The overall increase in temperature ranged from 5 to 10 °C for both measuring locations when increasing time exposure from 30 s to 60 s.

All samples exhibit excellent thermal insulation properties. Consequently, a general observation can be made regarding the temperatures measured at both locations (contact points situated on the rear walls of the second and third air chambers, as viewed from the front of the sample, as shown in Figure 1), and that is none of the tested configurations caused the recorded temperature to rise above 50 °C, comfortably below the maximum allowable limit of 180 °C for the TPS back-face temperature, thus ensuring the spacecraft and its components remain within design integrity.

## 4. Short Discussion

The newly developed air chamber architecture, based on structural thermal management by forming small internal air pockets, combined with continuous fiber reinforcement and surface coatings both functioning as a thermal and abrasion barrier, demonstrated strong potential for structural single-use components high-temperature ablative applications, with a focus on saving weight.

While the primary outcome of the present research focuses on thermal management through the novel internal air chamber architectural concept and the integration of 3D printing technology as a valuable tool for producing complex structures with reduced time and cost, the structural materials used in the additive manufacturing of the samples remain a key factor as well.

Overall, the developed 3D-printed configurations assured the necessary protection of the supposed underlying structure (backside of the samples) from excessive heat by dissipating heat through melting, vaporization, and decomposition (pyrolysis) upon exposure to high temperatures (1450 ± 50 °C), since the recorded temperature in two rear locations of the samples did not exceed 50 °C. Nevertheless, to gain a deeper understanding of the erosion or ablation mechanisms in the newly developed configurations and to ensure their controlled production, further studies are required.

The results clearly indicate that the reinforcement phase within the developed fiber-reinforced 3D printed composites plays a critical role in both heat transfer mechanisms and maintaining the structural integrity of the samples during OTB ablation testing.

## 5. Conclusions and Further Outlook

Two continuously reinforced configurations, 4 (carbon) and 5 (glass), demonstrated superior thermal stability, displaying the lowest thickness reductions, mass loss, and recession rates. Both configurations experienced approximately half the mass loss of the other tested configurations, ranging from around 5% after 30 s of exposure to 10–12% after 60 s, consistent with the trends observed in thickness loss. Among them, configuration 5, reinforced with continuous glass fibers, showed the lowest mass loss across both exposure durations, benefiting from the intrinsic properties of glass, namely its non-combustibility, low thermal conductivity, and high abrasion resistance. Notably, the Al_2_O_3_ surface-coated configuration 1 displayed mass loss values comparable to the reinforced configurations, suggesting that improved surface coating adhesion could offer a promising avenue for enhancing thermal protection performance. Although mechanically, hybrid configurations 2 and 3 revealed higher mass loss and recession rates, all the developed configurations showed a remarkably high thermal resistance. This provides evidence of the benefits offered by the novel air chamber architecture in terms of thermal management and underlying structure protection, achieved through reduced thermal conductivity.

Consequently, two newly developed ablative TPS configurations featuring an internal air chamber concept, produced via additive manufacturing, showed promising thermal stability and ablative characteristics results. The results clearly demonstrate that the reinforcement phase within the developed fiber-reinforced 3D-printed composites plays a crucial role in governing heat transfer mechanisms and preserving the structural integrity of the samples during OTB ablation testing. The use of 3D-printing technology in the development of ablative TPS composites enabled the development of complex geometries and structural architectures with minimal time and cost resources, offering the potential for in situ manufacturing in space.

A clear direction for optimizing configurations 4 and 5 involves increasing the volume fraction of the continuous reinforcement phase in both the frontal and radial walls, potentially reducing the frontal wall thickness, and exploring the possibility of decreasing the number of air chambers in the structural architecture while still enhancing ablative resistance. A second path for future research is to further develop hybrid configurations, such as 3, by combining the advantages of CFRP thermoset with 3D-printed thermoplastic composites TPS structures and ultimately incorporating a protective surface coating. Both future research directions shall exploit advanced 3D printing techniques to enable the fabrication of intricate geometries with customized thermal and mechanical performance characteristics.

## 6. Patents

A national patent request was filed prior to the present work in relation to the new internal air chamber concept of a heat shield obtained through additive manufacturing with low weight reference and air/vacuum cushions intended for space applications, Teodor-Adrian Badea, Alexa Crisan, and Raluca Maier, reference no. A/00073 OSIM: 26 February 2025.

## Figures and Tables

**Table 1:** Specifications and nomenclature of the newly fabricated composite ablative TPS samples, additively manufactured with the integrated internal air chamber architecture.

Material	Configuration Code	Sample Code/Mass *			
		Sample (30 s)	Mass [g] *	Sample (60 s)	Mass [g] *
Onyx FR ^1^	Al_2_O_3_-SC	1.1	43.981 ± 0.003	1.2	43.977 ± 0.003
	Al_2_O_3_-FS 5g	2.1	39.168 ± 0.003	2.2	39.173 ± 0.003
	CFRP-Disk	3.1	39.543 ± 0.003	3.2	39.469 ± 0.003
	CCF-F	4.1	44.649 ± 0.003	4.2	44.629 ± 0.003
	CGF-F	5.1	48.902 ± 0.003	5.2	50.452 ± 0.003

^1^ FR—Fire resistant; Al_2_O_3_ powder SC-surface coating; Al_2_O_3_ FS powder 5 g filled slot; CFRP-disk—mechanically integrated CFRP disk laminate; CGF-F—continuously glass fiber reinforced frontal wall area; CCF-F—continuously carbon fiber reinforced frontal wall area; * mass weight were determined prior to tests (excluding fixation parts).

**Table 2:** Recession rates and cross-section view of tested configurations.

30 s/Total Erosion (mm)	Erosion Rate (mm/s)	60 s/Total Erosion (mm)	Erosion Rate (mm/s)
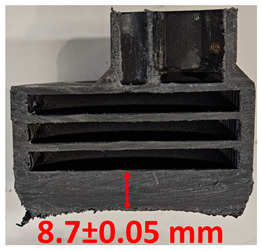	0.056 ± 0.002 mm/s	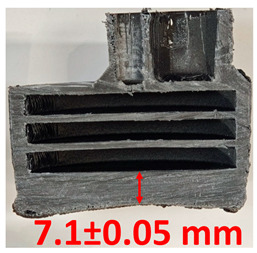	0.055 ± 0.002 mm/s
1.1		1.2	
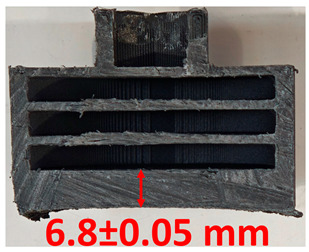	0.12 ± 0.002 mm/s	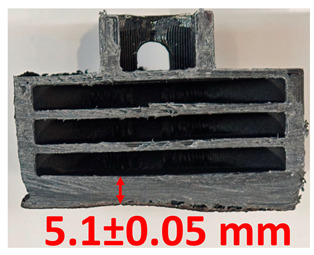	0.088 ± 0.002 mm/s
2.1		2.2	
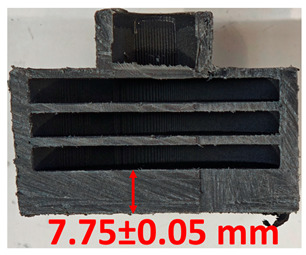	0.088 ± 0.002 mm/s	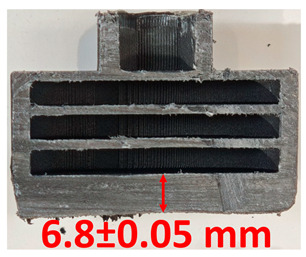	0.06 ± 0.002 mm/s
3.1		3.2	
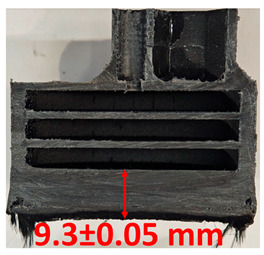	0.036 ± 0.002 mm/s	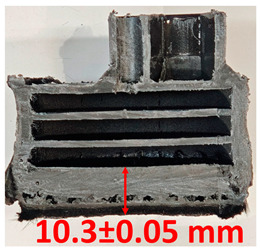	0.001 ± 0.002 mm/s
4.1		4.2	
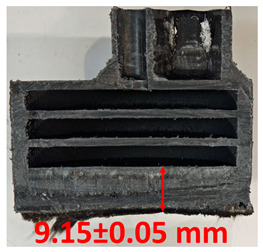	0.041 ± 0.002 mm/s	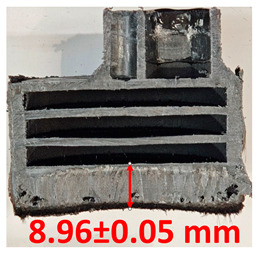	0.024 ± 0.002 mm/s
5.1		5.2	

## Data Availability

The original contributions presented in the study are included in the article, further inquiries can be directed to the corresponding author.
